# Identification of Auxin Response Factor-Encoding Genes Expressed in Distinct Phases of Leaf Vein Development and with Overlapping Functions in Leaf Formation

**DOI:** 10.3390/plants8070242

**Published:** 2019-07-23

**Authors:** Mathias Schuetz, Mario Fidanza, Jim Mattsson

**Affiliations:** 1 Department of Biological Sciences, Simon Fraser University, 8888 University Drive, Burnaby, BC V5A 1S6, Canada; 2 Department of Botany, The University of British Columbia, 6270 University Boulevard, Vancouver, BC V6T 1Z4, Canada; 3 Department of Neurosurgery, Stanford University, 300 Pasteur Dr., Palo Alto, CA 94304, USA

**Keywords:** MONOPTEROS, auxin response factor, ARF, shoot meristem, leaf initiation, vascular, procambium, Arabidopsis

## Abstract

Based on mutant phenotypes the *MONOPTEROS (MP)/Auxin Response Factor 5 (ARF5)* gene acts in several developmental processes including leaf vein development. Since overlapping functions among *ARF* genes are common, we assessed the related *ARF 3-8* and *19* genes for potential overlap in expression during vein development using in-situ hybridization. Like *MP/ARF5, ARF3* was expressed in preprocambial and procambial cells. *ARF7* was also expressed in procambial cells, close to and during vein differentiation. *ARF19* was expressed in differentiating vessel elements. To assess if genes with vein expression have overlapping functions, double mutants were generated. While *arf3, 5* and *7* mutants formed leaves normally, double mutant combinations of *mp/arf5* with *arf3* or *arf7* resulted in a breakdown of leaf formation. Instead, novel structures not present in any of the single mutants formed. The results implicate *ARF3* and *ARF7* in rosette leaf formation and suggest that their functions overlap and act in parallel with *MP/ARF5* in this process. The observed vascular expression patterns suggest unique functions (*ARF7* and *19*) and potentially overlapping functions (*ARF3* and *5*) in vein development. Since *arf3 arf5* double mutants do not form leaves, assessment of their potential combined action in vein development will require the use of conditional mutants.

## 1. Introduction

The phytohormone auxin has been implicated in a diverse set of plant developmental processes ranging from pattern formation in embryogenesis to gravitropism [1]. Molecular genetic and cell biological studies in the model plant *Arabidopsis thaliana* have resulted in the identification of important components of auxin signal transduction. Auxin is perceived by the TIR1/AFB and AUX/IAA families of auxin co-receptors [2,3]. TIR1/AFB proteins are, in turn, associated with the SCF (Skp1-Cul1-F-box) ubiquitin E3 ligase enzyme complex. Auxin binding to the TIR1/AFB AUX/IAA co-receptor proteins leads to the ubiquitination and degradation of AUX/IAA proteins, which are negative regulators of auxin responsive gene transcription [4,5,6,7]. Since AUX/IAA proteins dimerize with, and thereby inhibit, the transcription factor activity of Auxin Response Factors (ARF), the degradation of AUX/IAA proteins results in the de-repression of ARF activity and leads to auxin-responsive gene activation or suppression. The majority of AUX/IAA-ARF interactions are thought to occur via a shared C-terminal dimerization motif (PB1 domain) and some examples of ARF hetero and homo-dimerization have also been documented [8,9,10]. This simple pathway presumably produces a multitude of outputs through diverse expression patterns and/or functions of the many members of the *TIR1/AFB, AUX/IAA* and *ARF* gene families [10,11,12]. *ARF5-8* and *ARF19* have been implicated as transcriptional activators and the rest are considered to function as transcriptional repressors (reviewed in [2]), although there is evidence that *ARF7* and *ARF19* can act as both activators and repressors depending on the target genes [13], and similar conclusions may well eventually be reached for other ARF-encoding genes.

Phylogenetic analysis of the 23 annotated ARFs in the *A. thaliana* genome reveals clades as well as sister pairs. Sister pair double mutants have in several instances revealed phenotypes where single mutants do not, or stronger double mutant phenotypes than corresponding single mutants [2,13]. This genetic redundancy is linked to overlapping expression patterns [11]. Based on mutant phenotypes, the *ARF2* gene promotes progression through developmental stages, and its sister gene *ARF1* acts in part redundantly in these processes [14,15]. *ARF1* and *ARF2* also act redundantly with *ARF6* in embryo development [11]. *ARF3*/*ETTIN* plays a role in flower development [16,17]. While *arf3* mutant leaves are normal, *arf3* and *ar4* double mutants develop leaves with abaxial to adaxial tissue transformation [18]. *ARF3* and *ARF4* are also regulated by the same siRNA [19,20]. *ARF8* inhibits fruit development in the absence of fertilization [21,22] , and regulates petal, hypocotyl and root growth [23,24,25]. *ARF8* acts with its sister gene *ARF6* to promote jasmonic acid production and flower maturation [26] and are regulated by the same microRNA [27]. *ARF7/MSG2/NPH4* mediates phototropism signaling [28,29,30] and acts redundantly with the ARF19 sister gene to promote leaf expansion and lateral root formation [31]. ARF19 activity in root formation is modulated by phosphate availability [32], providing a glimpse into potential ARF roles in environmental regulation of growth. *The ARF5/MONOPTEROS (MP)* gene lacks a sister gene in the *A. thaliana* genome [13,33]. *Arf5/mp* mutants fail to form an embryonic root and hypocotyl and typically have a defective number and spacing of cotyledons [34]. When seedlings are rooted in vitro and transferred to soil, a rosette of leaves is formed, but the inflorescence stems fail to form flowers [35]. Cotyledons and leaves of *arf5/mp* mutants have a much-reduced venation pattern [35,36]. The expression of *ARF5*/*MP* and *ARF7* overlap partially during embryogenesis, and double mutant populations show an enhanced frequency of seedlings without cotyledons [9], providing evidence for a partial functional redundancy outside of an *ARF* sister pair. The role of *ARF5/MP* in embryonic patterning and its molecular interactions have been studied in some detail [11,37,38,39,40,41]. During leaf development, *ARF5/MP* expression is initially wide and gradually restricted to sites where veins form [42]. This expression is companioned by an *ARF5/MP*-dependent expression of *PIN-FORMED1* (*PIN1)* [42,43], an auxin efflux carrier, consistent with a role in a canalization of auxin flow mechanism that results in strands of interconnected vascular cells [42,43,44]. 

The *arf5/mp* mutant phenotype is enigmatic in the sense that mutants form one type of lateral organs (leaves) but not another (flowers). Similarly, mutant leaves lack much but not all venation. To test the hypothesis that other *ARF* genes may act redundantly with *ARF5/MP* in these processes, we examined phylogenetically related *ARF* genes for overlapping expression during leaf initiation and leaf development followed by double-mutant analysis of co-expressed *ARF* genes.

## 2. Results

Phylogenetic analysis shows that among the 23 *A. thaliana* ARF proteins, ARF 5, 6, 7, 8 and 19 are in the same clade [13,33,45]. In one study, ARF3 and 4 also fell in this clade [45] and were, therefore, included in this analysis. To assess if the expression patterns of these related *ARFs* overlap during leaf development, we used whole mount in-situ hybridization with antisense probes. As a negative control, we repeatedly hybridized with sense *ARF4* probes, revealing no unspecific background hybridization (Figure S1). Real-time quantitative PCR (RT-qPCR) confirmed that these genes are expressed in leaf primordia (Figure S2). For simplicity, we refer to immunolocalization of RNA hybrids indicative of enhanced steady-state levels of corresponding transcripts simply as “expression” and refer to genes with both mutant and numbered ARF names by their ARF name only. 

### 2.1. Differential Expression of ARF Genes during Leaf Development

**Figure 1:**
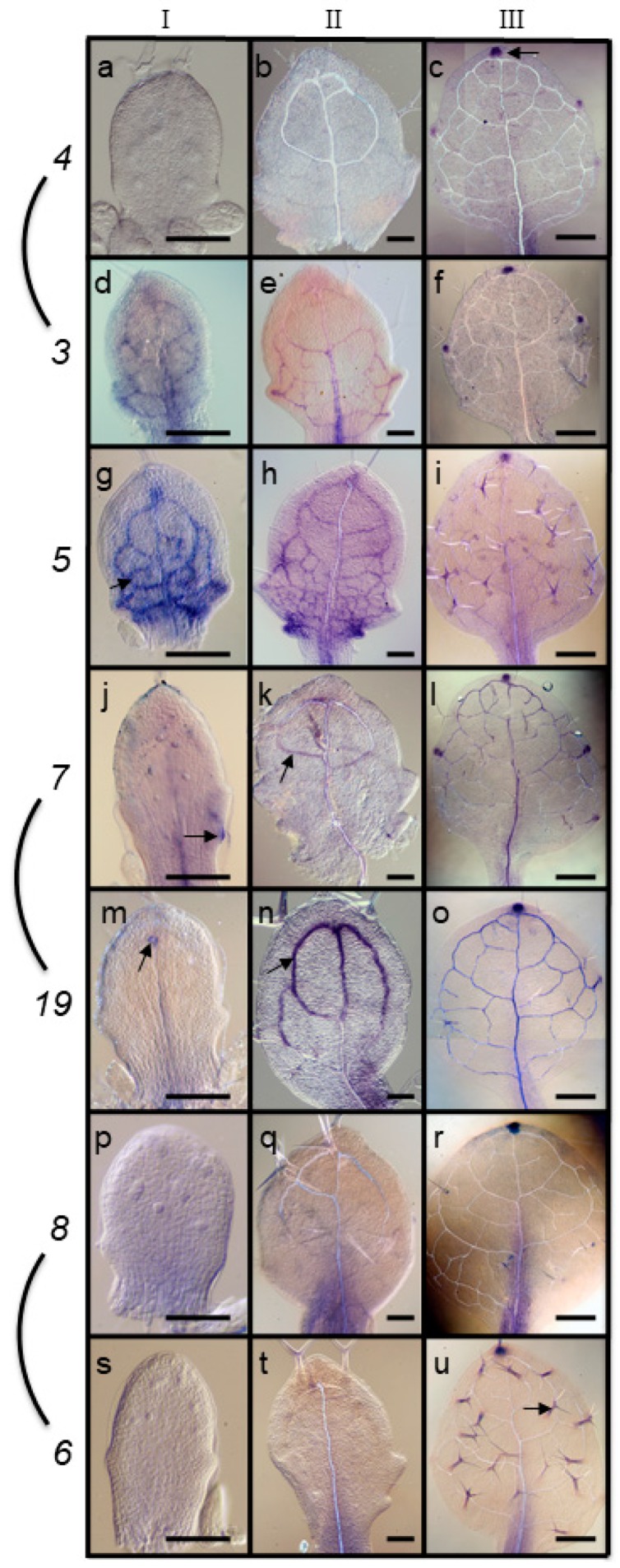
*ARF* gene expression patterns in developing leaves (blue/purple stain). (**a**–**c**) *ARF4*, (**d**–**f**) *ARF3*, (**g**–**i**) *ARF5/MP*, (**j**–**l**) *ARF7*, (**m**–**o**) *ARF19*, (**p**–**r**) *ARF8*, (**s**–**u**) *ARF6*. Arrows indicate examples of expression in apex hydathode (**c**), preprocambial/procambial (**d**), procambial cells undergoing xylogenesis (**m,n**) and trichome (**u**) cells. Size bars, 50 µm (**a**,**b**,**d**,**e**–**h**,**j**,**k**,**m**,**n**,**p**,**q**), 300 µm (**c**,**f**,**i**,**l**,**o**,**r**,**u**).

We assessed three stages of immature leaves: (I) Rich in pre-procambial and procambial cells (column I, Figure 1); (II) distal half containing differentiating vessel elements, proximal half containing procambial cells; and (III) whole leaf containing differentiating vessel elements. The experiments revealed dynamic expression patterns (Figure 1).

As described previously [42], *ARF5* is expressed in preprocambial and procambial cells (Stage I, Figure 1f). *ARF5* is also expressed in veins undergoing xylem and phloem differentiation (distal half, Figure 1h), but expression is weak or absent in differentiated veins (Figure 1g). At this stage, *ARF5* is also expressed in the developing apical and lateral hydathodes at leaf blade serrations and in the petiole.

A comparison of hybridization results in stage I leaf primordia shows that only *ARF3* has a procambial expression comparable to *ARF5* (Figure 1d). At this stage, both *ARF 3* and *5* are also expressed in cortex cells between procambial veins and close to the margin in the basal half of primordia. *ARF7* and *ARF19* show weak expression only in the procambial midvein, the oldest portion of the venation (Figure 1j,m). *ARF4, 6,* and *8* show no hybridization in stage I primordia (Figure 1a,p,s). A similar pattern of expression can be seen in stage II primordia. *ARF3* is expressed primarily in procambial cells in the basal half of the primordia, including procambial hydathodes (Figure 1e), which partially overlaps with *ARF5* expression (Figure 1h). The sister pair *ARF7* and *19* also showed vascular expression in stage II primordia, but in the apical part of the primordia (Figure 1k,n), containing differentiating veins. The sister pair *ARF6* and *8* are also expressed in stage II primordia in a wedge shape, wide at the petiole and narrowing apically towards the midvein (Figure 1q,t). *ARF5, 7* and *19* also showed diffuse midrib expression in the basal portion of stage III primordia (Figure 1l,o). As illustrated by Figure 1l,o, the sister pair *ARF7* and *19* are expressed in many of the veins of stage III primordia. Otherwise the unifying aspect in stage III primordia was a strong expression in the apical hydathode, with variable degree of expression in lateral hydathodes (Figure 1c,f,i,l,o,r,u). Finally, *ARF6* and *ARF5* expression was also observed in trichomes in stage III leaves (Figure 1i,u).

### 2.2. Leaf Anlagen and Preprocambial Expression of ARF3

**Figure 2:**
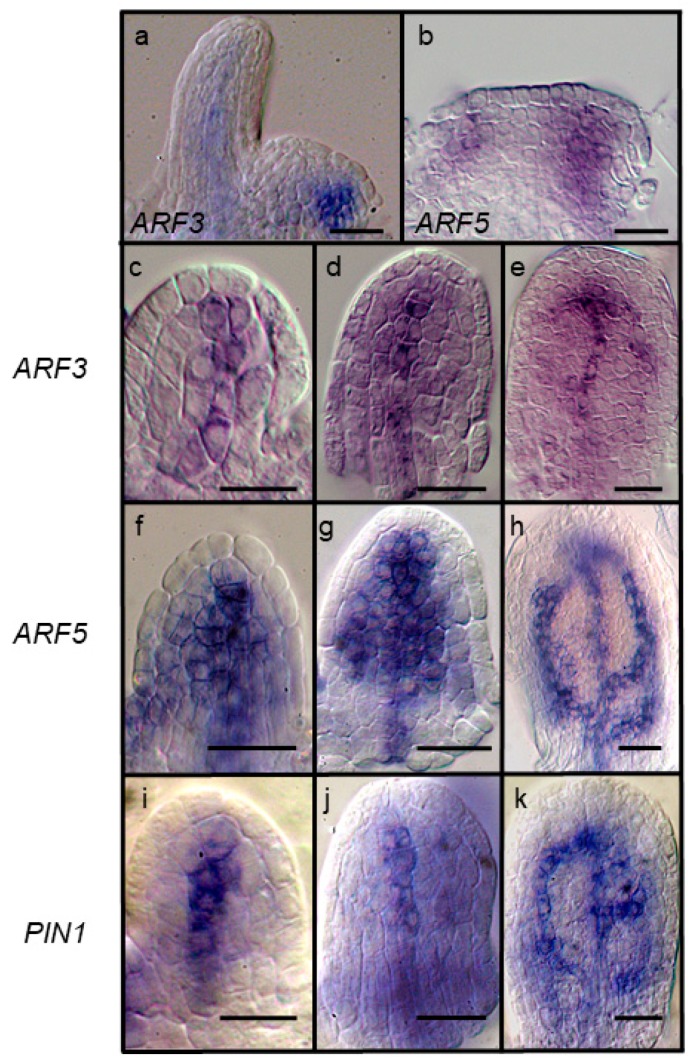
Expression of ARF3, ARF5 and PIN1 during early stages of leaf development. *ARF3* (**a**) and *ARF5* (**b**) expression is observed at the flanks of the SAM prior to leaf initiation. Stage 1 (**c,f,i,l**), between 1 and 2 (**d,g,j**) and 2 (**e,h,k**) leaf primordia showing expression of *ARF3* (**c**–**e**), *ARF5* (**f**–**h**), *PIN1* (**i**–**k**). Size bars, 20 µm.

Among the *ARF* genes tested here, *ARF3* showed an expression pattern most like *ARF5* during vein formation (compare Figure 1d,e to Figure 1g,h). ARF 3 and 5 also share a similar expression in leaf anlagen (Figure 2a,b). We compared the expression of *ARF3* to two preprocambial and procambial markers, *ARF5* and *PIN1* [42,43,46,47] in three early stages based on *ARF5* expression: Preprocambial midvein expression (Figure 2a); wide *ARF5* expression preceding formation of first pair of secondary veins (Figure 2b); and the appearance of the next set of secondary preprocambial veins (Figure 2c). *PIN1* shows a pattern similar to ARF5 at these stages, except that the zones of expression are narrower than *ARF5* (Figure 2i–k) [42]. Early *ARF3* preprocambial midvein expression (Figure 2c) is not as wide as *ARF5* and more like *PIN1*. At the next stage, *ARF3* transcripts are detected only in the midvein region (Figure 2d) like *PIN1*. At the third stage, *ARF3* transcripts are detected in the procambial midvein and portions of first pair of secondary veins (Figure 2e), which is less extensive than both *ARF5* and *PIN1*.

### 2.3. Expression of ARF7 and 19 in Differentiating Vessel Elements

**Figure 3:**
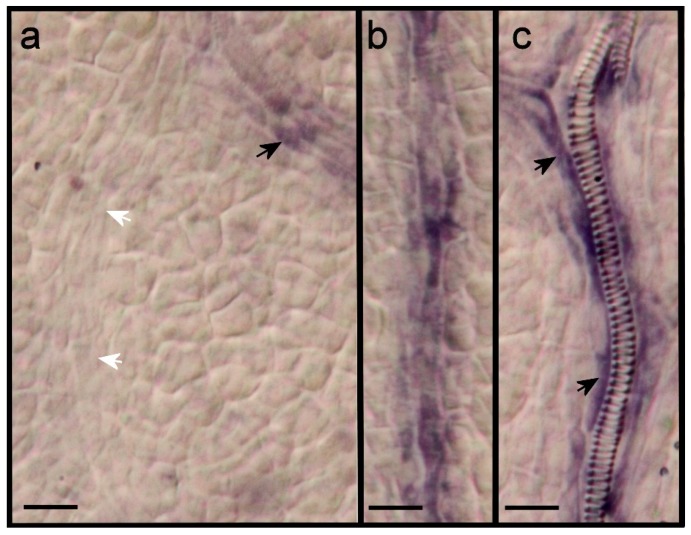
Expression of *ARF7* during vein differentiation. (**a**) Expression in older procambial secondary vein (black arrow) but not in younger, more basal, procambial secondary vein (white arrows), (**b**) expression in procambial secondary vein, (**c**) expression in elongated cells flanking a differentiating vessel element (back arrows). Size bars, 20 µm.

**Figure 4:**
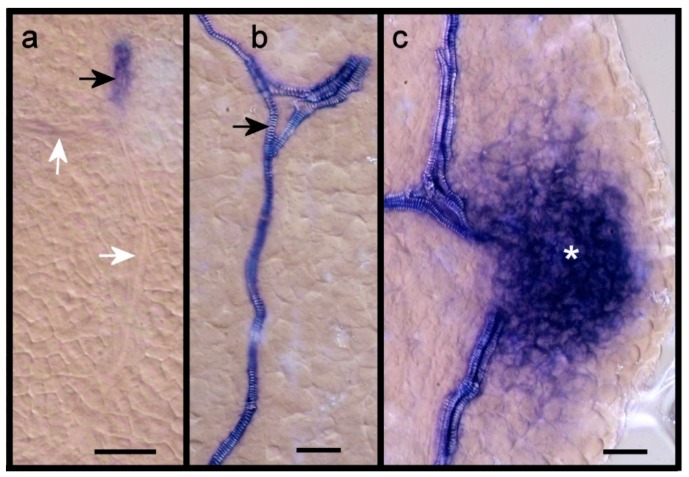
Expression of *ARF19* during vein differentiation. (**a**) Expression in differentiating vessel element extending towards leaf margin (black arrow) but not in younger procambial secondary veins (white arrows), (**b**) expression in differentiating vessel elements (black arrow), (**c**) expression in the hydathode region of a leaf serration (white asterisk). Size bars, 20 µm.

*ARF7* and *19* are expressed in veins of maturing leaves (Figure 1). We assessed this expression in additional primordia and at a higher magnification. *ARF7* is expressed in older procambial strands but not in more recently formed procambial strands (Figure 3a,b). At the stage when spirally lignified vessel elements appear, the expression occurs in flanking elongated cells that may still be procambial, but not in the differentiating vessel elements (Figure 3c). *ARF19*, on the other hand, is expressed in differentiating vessel elements (Figure 4a–c). Like *ARF7*, *ARF19* is expressed in parenchyma cells in hydathode regions (Figure 4c), especially where serrations develop. 

### 2.4. Auxin Induction of ARF Gene Transcription

**Figure 5:**
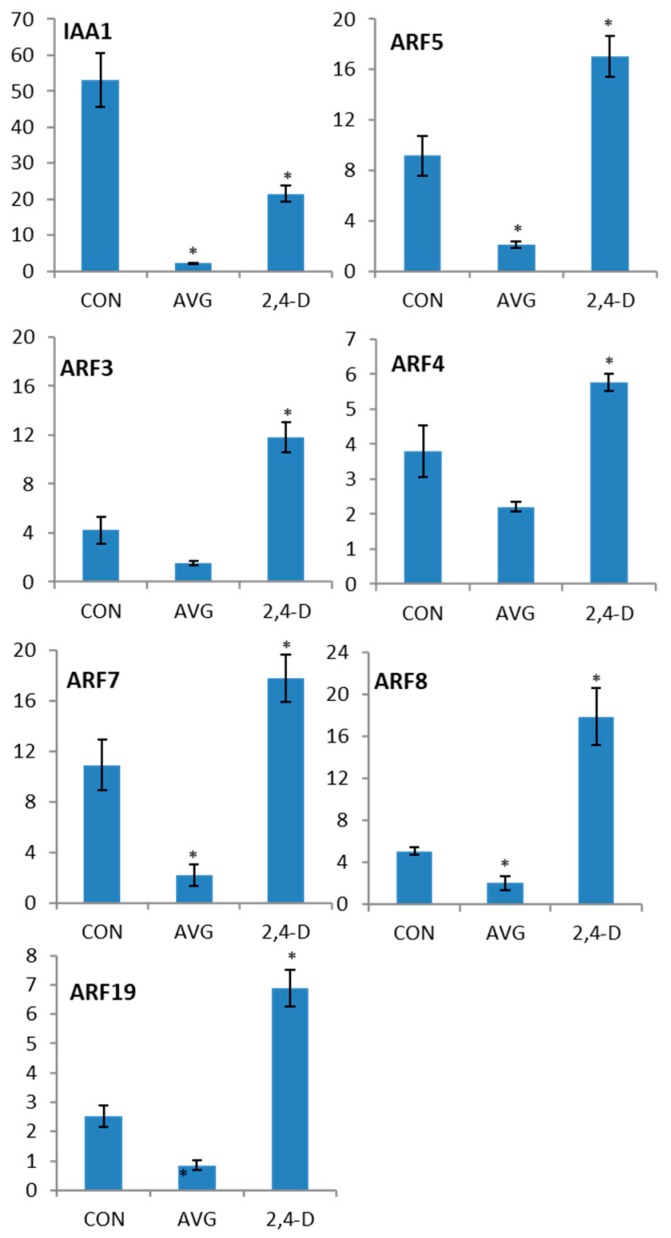
Auxin regulation of *ARF* gene transcript levels. The left bar indicates expression in untreated control seedlings, the middle bar indicates expression in seedlings after a 28 h AVG treatment, and the right bar 28-h AVG treatment followed by 4 h of 2,4-D treatment. All values on the y axes are expressed in terms of transcript abundance relative to the control gene *APT1*. IAA1 is used as a positive control based on well-known auxin inducibility. Student’s T-test, significant *p*-values (<0.05) are indicated by asterisks. Error bars represent standard deviations.

ARFs are known to be post-translationally activated by auxin [2]. In addition, two reports have shown an increase in *ARF5* transcript levels in response to exogenous applied auxin [42,48]. The diverse expression patterns observed above indicate different regulations of transcription. To test if auxin-activated transcription, direct or indirect, could be part of the regulation of these genes, we first used aminoethoxyvinylglycine (AVG), an auxin biosynthesis inhibitor that has been shown to significantly reduce endogenous auxin concentration in *A. thaliana* seedlings [49]. RT-qPCR analysis of AVG-treated seedlings indicated a significant decrease, ranging from 1.6- to 5-fold down regulation, in the transcript abundance of all tested *ARF* transcripts (Figure 5). The growth media of parallel-grown AVG treated seedlings was supplemented with the synthetic auxin 2,4-D to assess induced expression. After a four-hour 2,4-D treatment the transcript abundance of *ARF 3, 4, 5, 7, 8* and *19* significantly increased to either meet or exceed the expression levels of the controls (Figure 5). These results show that after AVG treatment all tested ARF genes responded to 2,4-D treatment with significantly higher transcript levels, with the highest levels for *ARF 3, 5* and *8* and lower levels for *ARF 4, 7* and *19*.

### 2.5. Response of arf Mutants to Auxin Transport Inhibition

**Figure 6:**
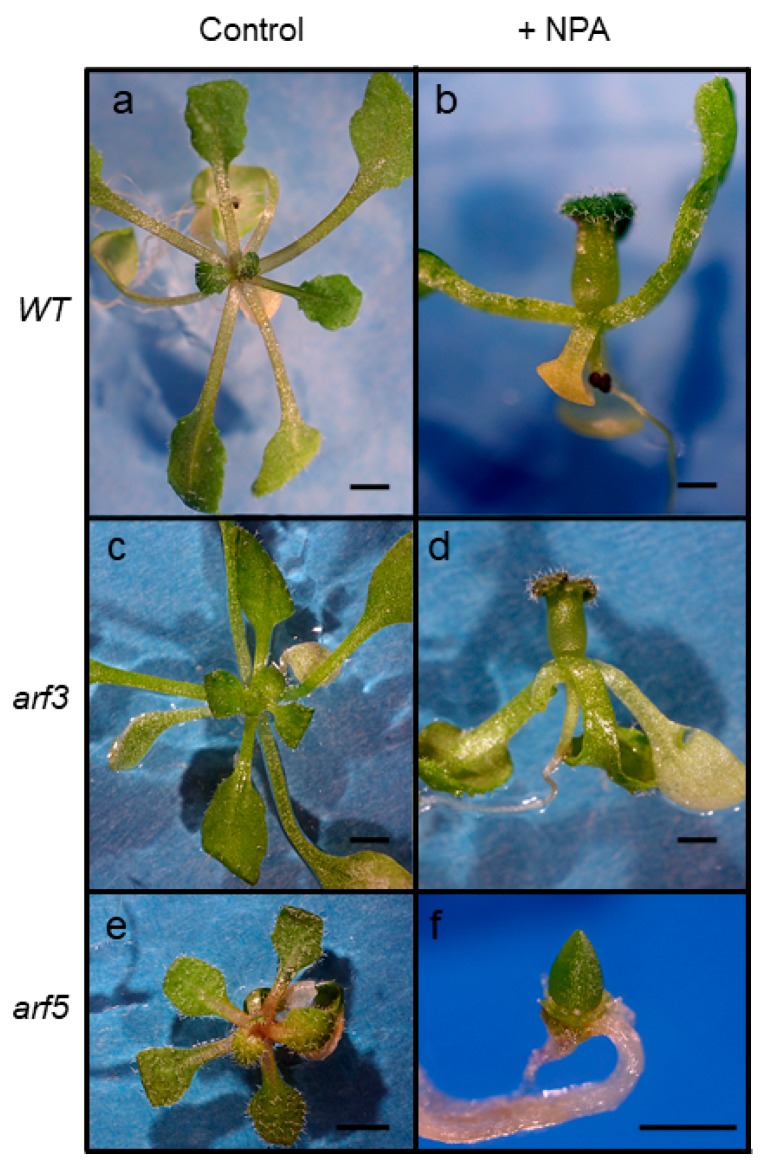
Leaf initiation in NPA-treated *arf* mutants. WT (**a**), *arf3* (**c**) and *arf5* (**e**) rosette of leaves when grown under control conditions. When grown in the presence of 10 µM NPA, WT (**b**), *arf3* (**d**), *arf7* and *arf19* mutants still produce leaves and usually terminate with a tubular leaf (data not shown). *mp/arf5* mutants cease forming leaves in response to NPA, resulting in a leafless dome (**f**). 21 DPI, size bars are 0.5 mm.

*Arabidopsis thaliana* seedlings respond to auxin transport inhibitors by forming wider leaf primordia, and, at higher concentrations by forming a leaf primordium that encircles the meristem, resulting in a tubular leaf (Figure 6b). In contrast, *arf5* mutants respond to auxin transport inhibition, either genetic or pharmacological, by terminating leaf formation, resulting in the formation of one or more large leafless domes at the site of the shoot apical meristem [50]. We hypothesized that mutants in the closely related *ARFs* assessed here could be equally sensitive to auxin transport inhibition if they also play a role in leaf formation. Loss-of-function T-DNA insertion lines of *ARF 3, 4, 6, 8* and *19* genes [13] were grown on media supplemented with naphthylphtalamic acid (NPA) and analyzed for leaf initiation defects. Except for *arf5*, the *arf* mutants did not respond differently from wildtype plants upon NPA treatment and plants usually formed a tubular leaf like what is observed in *WT* plants when grown on NPA-containing medium (Figure 6; only *arf3* phenotype shown). The results indicate that in the presence of a functional MP/ARF5 protein, the process of leaf initiation is not disrupted by mutations in other closely related *ARF* genes under tested conditions of reduced polar auxin transport.

### 2.6. mp/arf5, arf7 and mp/arf5, arf3 Double Mutants Terminate Leaf Formation

To test if the functions of *ARF5* in leaf formation and vein patterning overlap at least in part with the *ARF* genes showing incipient primordia and vascular expression (3, 7 and 19), we generated double mutant combinations of *ARF5* with insertion mutants in these three genes. We were unable to recover *arf5 arf19* double mutants, probably because of the close physical distance between these genes on chromosome 1 (230 kilobase-pairs, TAIR).

**Figure 7:**
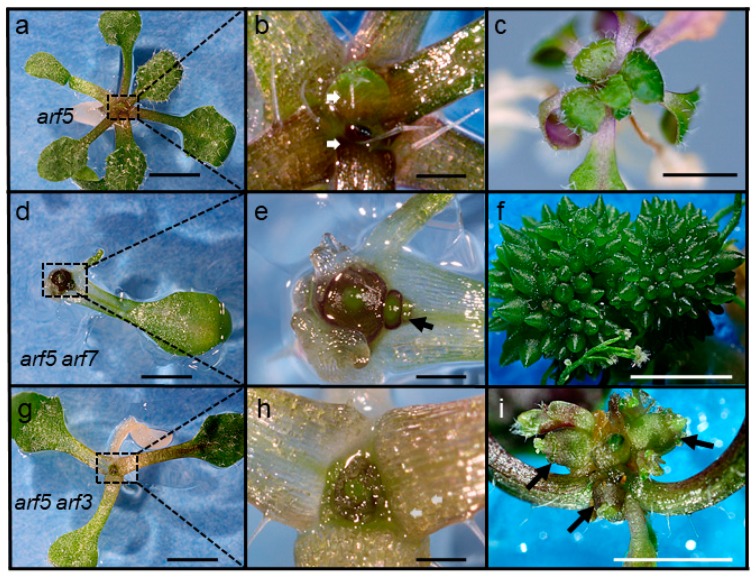
Leaf initiation defects in *arf5 arf7* and *arf5 arf3* double mutants. (**a**–**c**) *arf5*, (**d**–**f**) *arf5 arf7* double mutants, (**g**–**i**) *arf5 arf3* double mutants. Shoot apices from (**a**,**d**,**g**) are enlarged in (**b**,**e**,**h**). Ages are, 21 DPI (**a**,**d**,**g**), 40 DPI (**c**) and 90 DPI (**f**,**i**). Arrows indicate emerging leaf primordia (**b**), emerging lateral dome (**e**), dome-like structures (**i**). Size bars, 300 µm (**a**,**c**,**d**,**f**,**g**,**i**), 100 µm (**b**,**e**,**h**).

All *arf5 arf7* double mutants either did not form leaves or ceased leaf formation after initiation of one or two leaves, instead developing a dome-shaped enlarged leafless shoot structure (Figure 7d,e). In parallel, additional domes emerged at the base of the first dome (arrow in Figure 7e), a process that was repeated until elaborate structures comprised of many individual domes were produced (Figure 7f). 

Like *arf5 arf7* double mutants*, arf5 arf3* double mutants also displayed defects in leaf formation (Figure 7g,h), although they generally produced more leaf-like organs than *arf5 arf7* double mutants before ceasing leaf formation. Upon prolonged culture, *arf5, arf3* mutants also formed several leafless domes (Figure 7i) but did not form the large number of domes observed in *arf5 arf7* (Figure 4f) or in *arf5 pin1* mutants [50].

## 3. Discussion

### 3.1. Experimental Approach

In-situ RNA hybridization targeting mRNA transcripts of the native gene presumably records the output of all native cis-elements of the gene, local chromatin remodeling as well as the impact of microRNA-mediated transcript degradation, factors that affect ARF expression [19,51,52,53] but are difficult to fully account for with the alternative method of marker gene fusions in transgenic plants. Nevertheless, it should be kept in mind that our approach results in extremely fragile leaf primordia, many of which break at different stages of the procedure, limiting the use of this method. The inconsistency in that we did not observe *ARF7* expression in incipient leaf primordia but observed an *arf5 arf7* mutant phenotype in leaf formation, also suggests that our in-situ hybridization is not sensitive enough to detect *ARF7* at sites of leaf formation. This is possibly due to the use of gene-specific RNA fragments that limit cross-hybridization but also results in reduced level of detection. The observed *ARF5* expression is comparable in pattern and levels to previous in-situ hybridizations [42] and translational *pARF5*:*ARF5:GFP* gene fusion [54] indicating sufficient detection of this gene. RT-qPCR from RNA of leaf primordia (Figure S2) show that tested *ARFs* may be expressed at lower levels than *ARF5*, so it is possible that we did not observe the whole picture of expression of these genes. 

### 3.2. Patterns of Overlapping Expression in Leaf Primordia

We hypothesized that members of the same clade as the *ARF5* gene may overlap with *ARF5* expression pattern during leaf and vein formation. Our in-situ hybridization data revealed that the expression of only *ARF3* overlapped with ARF5 during these processes. *ARF3* expression, however, appears to differ in that it does not show the wide domains of expression in cortical cells followed by narrowing to regions where procambial veins appear, characteristic of *ARF5* [42]. The *ARF5* expression dynamics has been suggested to reflect the process of canalization of auxin flow going from wide to narrow, and if that is the case, *ARF3* is not likely to play a role in this process or act late in it based on its narrow preprocambial and procambial expression. ARF5 is known to repress the expression of *ARR7* and *ARR15* in the peripheral zones of the floral meristem [55]. Since ARR7 and 15, in turn, are known to repress cytokinin signaling, ARF5 is thought to enhance cytokinin signaling at sites of expression in flower primordia [55]. Recently, ARF3 was shown to have the opposite function, that is, repress genes in cytokinin biosynthesis and signaling to regulate floral meristem maintenance [56]. If these functions were extended to the sites of leaf and vein formation, it would imply that *ARF5* and *ARF3* have opposing rather than, as hypothesized, overlapping functions, at least with respect to cytokinin signaling. Analysis of *ARF3* promoter-GUS fusions in advanced leaf primordia revealed expression in veins and in the cortex of growing leaf margins [53]. However, the histochemical assay for GUS activity is stronger, later and more extensive than the signals recorded by Pekker et al. [18] and by us. This discrepancy likely is at least in part due to the *TAS3*-mediated degradation of *ARF3* transcripts [20,57,58], which does not affect ARF3 promoter-GUS gene fusion. Fahlgren et al. [19] report the ectopic formation of leaf primordia on leaves from plants expressing a *TAS3*-insensitive *ARF3* gene. This finding supports a role for *ARF3* as a positive regulator of leaf primordia formation, which is consistent with the *ARF3* expression and *arf5 arf3* phenotype documented in this study. The expression of *ARF3* suggests that it may also play a role in vein development. However, *arf3* mutant leaves did not reveal any obvious phenotype, and *arf5 arf3* double mutants failed to produce leaves (see below), so there is no functional evidence at this point to support this hypothesis.

Members of two *ARF* sister pairs showed similar expression patterns. *ARF7* and *19* were expressed in differentiating veins. These two genes are known to act redundantly in lateral root development, stem growth, leaf blade expansion, and hypocotyl and root gravitropic responses [13,31,59], suggesting that they may act together also in the regulation of vein differentiation. Likewise, *ARF6* and *8* double mutants show defective and delayed flower development [13,60]. Both *ARF6* and *8* showed diffuse petiole and midrib expression, indicating that they may act together also in the development of these structures.

*ARF5* expression in leaf primordia is auxin inducible [42], consistent with the predicted positive feedback loop behind canalization of auxin flow during selection of cells for vein formation. There is limited evidence that expression of other ARFs are also upregulated in response to exogenous auxin [61]. However, since the tested genes showed quite different expression patterns in leaf primordia, we were surprised to see that the steady-state transcript levels of all tested genes were significantly reduced in response to the auxin biosynthesis inhibitor AVG [49] and many times higher after subsequent exposure to the synthetic auxin 2,4-D. It should be noted that AVG is also a well-known inhibitor of ethylene biosynthesis and that high concentrations of 2,4-D can induce ethylene biosynthesis [62]. However, while experiments using similar AVG and 2,4-D concentrations for treatments on *A. thaliana* seedlings did result in down and up-regulation of auxin-induced genes, respectively, they did not result in altered expression of ethylene-induced genes [49]. Nevertheless, we cannot rule out that at least part of the observed responses is ethylene related, especially as ethylene can modulate both auxin biosynthesis and signaling [63]. Although the auxin exposure in our experiments was short (4 h), it does not distinguish between direct or indirect activation of gene expression, or even regulation of transcript stability. Nevertheless, the results suggest that the diverse expression patterns are in part due to activation in regions of high auxin content and that tissue and region-specificities comes from different sets of other transcription factors and/or microRNA regulation. In support of a partial role of auxin activating the transcription of the tested genes, there is strong evidence for a role of auxin and polar auxin transport in preprocambial, procambial and vessel element differentiation [42,44,64], stages in which *ARF3, 7* and *19* are expressed. Additionally, all tested genes are expressed in hydathodes, regions known to express genes encoding proteins in auxin biosynthesis, many known auxin-regulated genes, and marker genes driven by synthetic auxin response elements [65,66,67]. Since the *ARF* genes are expressed strongly in fully differentiated hydathodes, the expression may have no role in hydathode formation. Instead, auxin flowing from hydathodes may support developmental and tropical responses along the route of polar auxin transport.

### 3.3. ARF7 and ARF3 Act Synergistically with ARF5 in Leaf Formation

Of the tested double combinations, *arf7 arf5* and *arf5 arf3* double mutants showed enlarging meristem regions coupled with failure to form leaves. While these drastic phenotypes precluded analysis of interactions in vein formation, they do shed some novel light on functions in the shoot apical meristem (SAM). First, as these phenotypes were not present in any of the single mutants, they indicate synergistic interactions and that these gene combinations act in parallel to maintain meristem organization and induce leaf formation. The cessation of leaf formation is perhaps not surprising considering that *arf5 arf7* embryos frequently do not form cotyledons [9], but not given, considering that cotyledons are formed during embryogenesis. The obtained double mutant phenotypes indicate that *ARF3* and 7 play a role in leaf formation, but that these functions are normally masked by *ARF5*, synergies typically found in *ARF* sister pairs.

## 4. Materials and Methods

### 4.1. Plant Material and Growth

The *mp/arf5^G12, G33, BS1354^, arf4-2, arf3-1, arf7-1, arf7^nph4-1^, arf19-1, arf8-2, arf6-1* mutant alleles used for double mutant and single mutant analysis have been described previously [13,29,36,68]. Surface sterilized seeds were plated on solid *Arabidopsis thaliana* salts media (ATS) [69]. For the auxin transport inhibitor treatments, seedlings were plated on media containing 0.1, 1 or 10 μM NPA (TCI, Tokyo, Japan). Seeds were imbibed and stratified at 4 °C for at least two days and grown in a short-day chamber (8 h light/16 h dark) at 20 °C and approximately 50 μEinstein light intensity. Time points for harvest and observations are indicated in Days Post Imbibition (DPI) when plates were transferred from 4 °C to growth conditions. 

Since *arf5* mutants are sterile, heterozygous *arf5* mutant plants were crossed with homozygous *arf3, arf7* and *arf19* mutants. In F2 and subsequent generations, homozygous *arf3* mutants were identified based on gynoecium defects and short siliques [35], and homozygous *arf7* and *arf19* mutants were identified based on narrower and epinastic rosette leaves compared to wildtype plants [13,30]. Populations homozygous for *arf3, arf7* or *arf19* were screened for segregation of *arf5* mutants lacking hypocotyl and root. At least ten independent homozygous *arf3* or *arf7* mutant plants which were segregating *arf5* mutants were identified and used for subsequent analysis. 

Approximately 80 putative *arf5 arf3* double mutants and over 200 *arf5 arf7* double mutants were isolated, transferred to new petri-dishes and grown in parallel with single *arf5* mutants. No populations segregating *arf5* in homozygous *arf19* mutant background were recovered, presumably due to the close linkage of the two loci (230 kb apart). Fifteen putative *arf5 arf3* double mutants were genotyped with respect to the presence of the *arf3-1* insertion mutant allele. All 15 plants were found to be homozygous for the *arf3-1* mutation, whereas *mp* and wildtype plants tested homozygous for the wildtype *ARF3* allele. ABRC stock number for *arf3-1* is CS24603, and the GenARF3_F (CCCATGGTGGTTTCTCTGTT) and GenARF3_R (CAGATGCAACTGCT GTGGTT) primers were used to confirm disruption of the *ARF3* gene while *mp/arf5* mutants were identified based on seedlings lacking a root and hypocotyl.

### 4.2. In-situ Hybridization and Molecular Biology

Genomic DNA for PCR genotyping assays was extracted using ChargeSwitch^®^ Plant genomic DNA extraction kit (Thermo-Fisher Scientific). cDNA was generated from total RNA extracted from 14 day old *WT* seedlings using Trizol reagent (Thermo-Fisher Scientific) and subsequently reverse transcribed using RevertAid™ M-MuLV Reverse Transcriptase (Thermo-Fisher Scientific) *ARF4*, *ARF19 and PIN1* fragments were amplified from cDNA using the primers *ARF19F* (CGCGCTCTCATCTTTTAACC), *ARF19R* (CCTCCACCATTCATGATTCC), *ARF4F* (AGGTTCTGCATCACCCTCAC) and *ARF4R* (TGCCTTTCTGTTTTCCCATC), *PIN1F* (TTTGTGTGGAGCTCAAGTGC), *PIN1R* (CTGCGTCGTTTTGTTGCTTA) using Phusion DNA polymerase (New England Biolabs). Fragments were cloned into pBluescript II (Stratagene). Plasmids containing cloned *ARF3* (U09387), *ARF6* (H7D8), *ARF7* (E11H5), and *ARF8* (M34A11) cDNAs were obtained from Arabidopsis Biological Resource Centre (ABRC) with the indicated stock number. All plasmids were sequenced to verify the cDNA sequence and determine insert orientation. In vitro transcription in the presence of DIG labelled UTP (Roche) was carried out using either T3 or T7 RNA polymerase (Thermo-Fisher Scientific) from linearized plasmid DNA. *ARF5* probes were generated as described [36]. Whole mount in-situ hybridization procedure was as described [70] but modified to include overnight fixation, agitation in a fresh solution containing 0.1 M triethanolamine (pH 8) and 0.5% (*v/v*) acetic anhydride for 15 min, followed by two washes in 1 × PBT solution prior to hybridization for two days at 60 °C. Each in-situ hybridization was carried out at least three times on freshly fixed *WT* or *arf5* plants. Roots, hypocotyls and mature leaves were removed from the shoot apical meristems and developing leaves, and 10–15 of these ‘immature’ leaf rosettes were used for each *in-situ* hybridization for each probe. Tissue samples were mounted in 30% glycerol or a solution containing chloral hydrate: glycerol: water 9:1:3 (*w/w/w*). DIC images were taken on a Nikon Eclipse 600 microscope using a Canon D30 digital camera. 

### 4.3. RT-qPCR Analysis of 2,4-D Induction of ARF Genes After AVG Treatment

*WT* seeds were planted in ½ MS liquid medium, stratified at 4 °C for 2 days, and transferred to a rotary shaker under 24 h light conditions. At 4 DPI, cultures were supplemented with AVG (aminoethoxyvinylglycine) to a final concentration of 40 M and incubated for 24 h on the shaker under constant light. A subset of cultures was then treated with 40 M 2,4-D. Whole seedlings from both pools were collected after 4 hours and RNA was extracted using Plant RNA Purification Reagent (Thermo-Fisher Scientific). cDNA was subsequently synthesized using AccuScript High Fidelity Reverse Transcriptase (Agilent). RT-QPCR was performed using a BioRad Real Time Quantitative Thermocycler and the DyNamo Flash SYBR Green qPCR Kit (Thermo-Fisher Scientific). Genes were amplified using the primers listed in Table 1. Amplification efficiency of primers were tested and found to be ≥1.9. Relative transcript abundance was then determined using the delta CT method and normalized to the *ADENINE PHOSPHORIBOSYL TRANSFERASE 1 (APT1)* gene [71]. 

## Figures and Tables

**Table 1:** DNA primer sequences used for RT-qPCR.

Gene	Forward Primer	Reverse Primer
*IAA1*	GGAAGAGAGCTTCTCCGTTAAA	CAGGAGGAGGAGCAGATTCTT
*ARF3*	TCATCACCCTCTTCCGTCTT	TTCCTTGCGAATGATGATGA
*ARF4*	TCCTGAAAAAGGATGGAGGA	AGGCTGGCTCACAGAAGATG
*ARF5*	GGGTCAGTCGGGAGATCAAT	CCTTACGCATCCCACAAACT
*ARF7*	AACTTTGCCGGTGTACCAGT	GCCCAGTGGAAACTTGAGAC
*ARF8*	CCATGGGAGTCATTTGTGAA	AGTGGAAACGACTTCAAATGG
*ARF19*	CCGCTAACCTCTGATTGGAA	AGCATTTCCGCTGTCTGTTT
*APT1*	TGGAAGGTTATTCGGAGGAG	AGGATCAAATCCCACGCAAA

## Supplementary Materials

The following are available online at https://www.mdpi.com/2223-7747/8/7/242/s1, Figure S1: Negative control in-situ RNA hybridization results using dig-labelled ARF4 sense strand transcripts as probes, Figure S2: Relative expression levels of assessed *ARF* genes and the *IAA1* gene in leaf primordia.
