# Supplementary material for: Identification of Auxin Response Factor-Encoding Genes Expressed in Distinct Phases of Leaf Vein Development and with Overlapping Functions in Leaf Formation
Source: Plants (Basel). 2019 Jul 23;8(7):242. doi: 10.3390/plants8070242 (PMC6681221; doi:10.3390/plants8070242)
Supplement: Supplementary file 1 [file plants-08-00242-s001.zip › Figure S1.pdf]

Identification of Auxin Response Factor-encoding genes expressed in distinct phases of leaf vein development and with overlapping functions in leaf formation

Mathias Schuetz, Mario Fidanza and Jim Mattsson

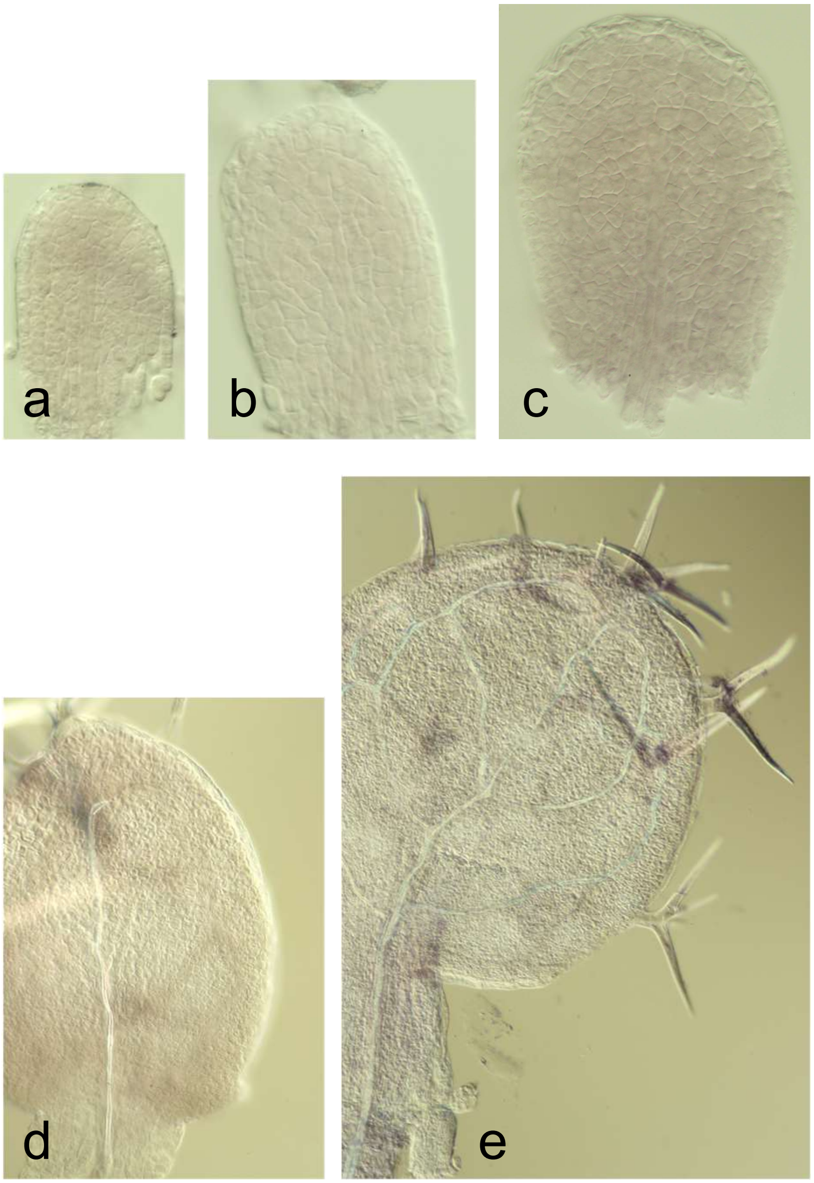

Figure S1. Negative control in situ RNA hybridization results using dig-labelled ARF4 sense strand transcripts as probes. No blue or purple color, indicative of hybridization, is detected. (a, b, c) stage I, (d) Stage II, (e) stage III primordia.
