# Supplementary material for: Identification of Auxin Response Factor-Encoding Genes Expressed in Distinct Phases of Leaf Vein Development and with Overlapping Functions in Leaf Formation
Source: Plants (Basel). 2019 Jul 23;8(7):242. doi: 10.3390/plants8070242 (PMC6681221; doi:10.3390/plants8070242)
Supplement: Supplementary file 1 [file plants-08-00242-s001.zip › Figure S2.pdf]

Identification of Auxin Response Factor-encoding genes expressed in distinct phases of leaf vein development and with overlapping functions in leaf formation

Mathias Schuetz, Mario Fidanza and Jim Mattsson

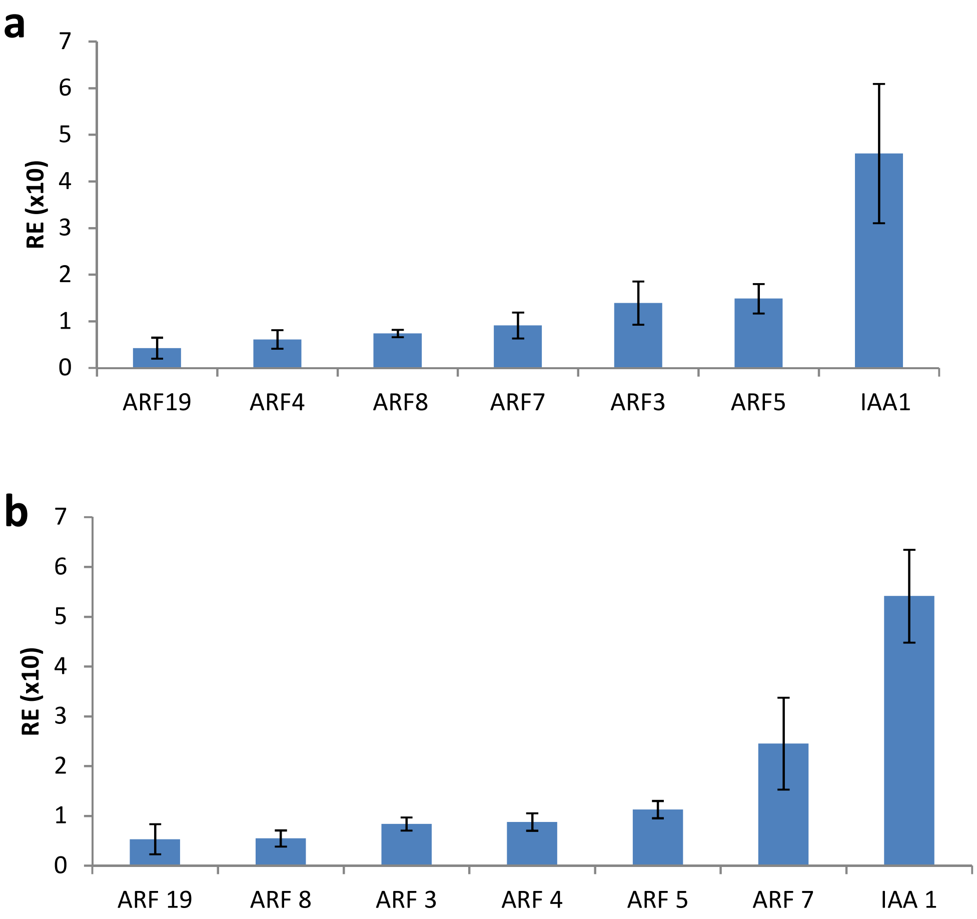

Figure S2. Relative expression levels of assessed *ARF* genes and the *IAA1* gene in leaf primordia. Leaf primordia of five day old **(a)** and six day old **(b)** seedlings were extracted, used for RNA purification, cDNA synthesis and RTQPCR as described in materials and methods. Relative expression was calculated using the delta CT method using the gene *ORNITHINE TRANSCARBAMYLASE* (OTC) gene (AT1G75330) as a normalization standard. Error bars represent standard deviation from three independent pools of extracted leaf primordia.
